# Potential of algae-derived alginate oligosaccharides and β-glucan to counter inflammation in adult zebrafish intestine

**DOI:** 10.3389/fimmu.2023.1183701

**Published:** 2023-05-19

**Authors:** Saima Rehman, Adnan H. Gora, Yousri Abdelhafiz, Jorge Dias, Ronan Pierre, Koen Meynen, Jorge M. O. Fernandes, Mette Sørensen, Sylvia Brugman, Viswanath Kiron

**Affiliations:** ^1^ Faculty of Biosciences and Aquaculture, Nord University, Bodø, Norway; ^2^ SPAROS Lda, Olhão, Portugal; ^3^ CEVA (Centre d’Etude et de Valorisation des Algues), Pleubian, France; ^4^ Kemin Aquascience, Division of Kemin Europa N.V., Herentals, Belgium; ^5^ Animal Sciences Group, Host Microbe Interactomics, Wageningen University and Research, Wageningen, Netherlands

**Keywords:** microalgae, macroalgae, prebiotics, metabolomics, RNA seq, β-glucans, gut

## Abstract

Alginate oligosaccharides (AOS) are natural bioactive compounds with anti-inflammatory properties. We performed a feeding trial employing a zebrafish (*Danio rerio*) model of soybean-induced intestinal inflammation. Five groups of fish were fed different diets: a control (CT) diet, a soybean meal (SBM) diet, a soybean meal+β-glucan (BG) diet and 2 soybean meal+AOS diets (alginate products differing in the content of low molecular weight fractions - AL, with 31% < 3kDa and AH, with 3% < 3kDa). We analyzed the intestinal transcriptomic and plasma metabolomic profiles of the study groups. In addition, we assessed the expression of inflammatory marker genes and histological alterations in the intestine. Dietary algal β-(1, 3)-glucan and AOS were able to bring the expression of certain inflammatory genes altered by dietary SBM to a level similar to that in the control group. Intestinal transcriptomic analysis indicated that dietary SBM changed the expression of genes linked to inflammation, endoplasmic reticulum, reproduction and cell motility. The AL diet suppressed the expression of genes related to complement activation, inflammatory and humoral response, which can likely have an inflammation alleviation effect. On the other hand, the AH diet reduced the expression of genes, causing an enrichment of negative regulation of immune system process. The BG diet suppressed several immune genes linked to the endopeptidase activity and proteolysis. The plasma metabolomic profile further revealed that dietary SBM can alter inflammation-linked metabolites such as itaconic acid, taurochenodeoxycholic acid and enriched the arginine biosynthesis pathway. The diet AL helped in elevating one of the short chain fatty acids, namely 2-hydroxybutyric acid while the BG diet increased the abundance of a vitamin, pantothenic acid. Histological evaluation revealed the advantage of the AL diet: it increased the goblet cell number and length of villi of the intestinal mucosa. Overall, our results indicate that dietary AOS with an appropriate amount of < 3kDa can stall the inflammatory responses in zebrafish.

## 1 Introduction

Inflammatory bowel disease (IBD) is a multifactorial disorder characterized by chronic and recurrent episodes of inflammation in specific segments of the intestine. IBD can be instigated by both genetic and environmental factors, but the rise in IBD cases over the last decade suggests the decisive role of diverse environmental factors in the pathogenesis of IBD (1). Moreover, the accelerated incidence of IBD in developing nations is correlated with a high intake of Western diet. Current approaches for the treatment of IBD include the use of different anti-inflammatory drugs (2). The remitting and relapsing nature of the disease necessitates prolonged use of such anti-inflammatory agents, leading to undesirable side effects (3). Diet is an important environmental factor that can be an alternative to drugs, since components such as prebiotics are known to regulate intestinal inflammation by maintaining immune homeostasis. These non-digestible carbohydrates are considered as establishers of beneficial bacteria that can produce bioactive metabolites, such as short-chain fatty acids that provide energy to enterocytes and maintain mucosal integrity (4).

Alginate oligosaccharides (AOS) are natural bioactive compounds and among other bioactivities have anti-inflammatory, antioxidant and prebiotic properties (5). They are produced through chemical or enzymatic digestion of alginates mainly extracted from brown algae. AOS are linear polymers of 2-25 monosaccharides composed of β-D-mannuronic acid (M) and α-L-guluronic acid (G) monomers linked by 1-4 glycosidic linkages with different M/G ratios and degrees of polymerization. The biological functions of AOS is dependent on the molecular weight (MW) (5). An *in vitro* study has reported that low molecular weight alginates enhance the radical scavenging and immunomodulatory capacities in the gut (6) and AOS < 1 kDa and 1.84 M/G can efficiently scavenge superoxide, hydroxyl, and hypochlorous acid radicals compared to AOS of MW 1 to 10 kDa (7). Furthermore, an *in vitro* study reported that AOS < 1kDa is effective in eliciting lysozyme activity, peroxidase activity, phagocytic capacity and total nitric oxide synthase activity compared to AOS of MW 1-2 kDa or 2-4 kDa (8). Inflammation suppressing ability of AOS has also been described previously; through attenuation of nitric oxide and prostaglandin E_2_ production and inactivation of the nuclear-factor kappa B and mitogen-activated-protein-kinase signaling pathways, as reported for mice macrophage cell lines (9) and enhancement of the activity of antioxidant enzymes such as superoxide dismutase (SOD) and catalase (CAT), as reported for human umbilical vein endothelial cells (10). Dietary AOS can also alter intestinal morphology and barrier function; by increasing the villi length, goblet cell number and mucin-2 (MUC2) expression (11). Furthermore, AOS supplemented diet ameliorated the inflammatory responses in a DSS-induced colitis mice model by reducing the infiltration of neutrophils and level of inflammatory markers (TNF-α, COX-2) and increasing the expression of tight junction proteins Zonula occludens-1 and Occludin (12). We have reported the ability of AOS to increase the abundance of bacteria associated with short chain fatty acid (SCFA) production (13). Most of the previous reports on the anti-inflammatory and antioxidant activities of AOS have been based on *in vitro* studies. Hence, it is essential to generate *in vivo* study-based evidence on the anti-inflammatory potential of AOS using an animal model. Furthermore, *in vivo* studies to understand the effect of molecular weight of AOS on its anti-inflammatory potential has not been explored in detail.

Algal β-(1, 3)-glucan is a known prebiotic derived from the unicellular alga *Euglena gracilis*. Paramylon is the storage polysaccharide in *E. gracilis* and it is a straight-chain β- (1, 3)-glucan (14). Previous studies have reported the anti-inflammatory potential of paramylon; oral administration of paramylon reduced the number of infiltrating CD3^+^ T-lymphocytes, and decreased expression of *Ccl2* and *Il-11* in the gut of gastric cancer mice model (14). Furthermore, paramylon treatment activated M2 macrophages and downregulated the expression of inflammatory cytokines in the liver of mice (15).

In the present study, intestinal transcriptome and plasma metabolome of zebrafish were profiled to reveal the effects of dietary AOS (*Laminaria* sp.-derived, with varying amounts of < 3kDa fraction). We employed an adult zebrafish intestine inflammation model to understand the efficacy of the macroalga-derived oligosaccharides to counter inflammation. In addition, we investigated the changes caused by AOS and those imparted by a well-known anti-inflammatory product, algal β-(1, 3)-glucan (16).

## 2 Materials and methods

### 2.1 Experimental fish

Adult zebrafish (8-month-old AB strain) were used for the experiment. To obtain this stock, the parents were bred in five tanks at the zebrafish facility of Nord University, Norway, following a previously reported protocol (17). Fifteen males and 30 females in each of the five replicate tanks were community bred to obtain 300-400 eggs from each tank. These eggs were kept in E3 medium and incubated at 28 °C in an incubator until hatching i.e., at around 50 h post-fertilization. Larvae at 5-day post -fertilization (dpf) stage were fed *ad libitum* commercial micro diets (< 100 µm particle size, Zebrafeed^®^, Sparos Lda, Olhão, Portugal). From 15 dpf (advanced larval stage), they were transferred to a recirculatory system and fed micro diets of 100-200 µm particle size (Zebrafeed^®^). From 30 days post-fertilization, the fish were fed a zebrafish diet (Zebrafeed^®^) of 300 µm particle size. Upon reaching the 8^th^ month, 250 male zebrafish weighing 300-400 mg were transferred to a freshwater flow-through system (Zebtec Stand Alone Toxicological Rack, Techniplast, Varese, Italy) and acclimatized in 3.5 L tanks of the system. These fish were randomly distributed into 25 tanks (10 fish per tank). The water temperature in the tanks was 28°C; the water flow rate was 2.5 L/h and dissolved oxygen in the tanks ranged between 7-8 ppm (oxygen saturation above 85%). A 14L:10D photoperiod was maintained throughout the 30-day feeding experiment.

### 2.2 Diet preparation and feeding experiment

Sparos Lda. prepared the five experimental diets (**Supplementary Table 1A**): One control diet and four soybean-based diets. The control (CT) diet was a fish meal-based diet with high-quality marine protein. Soybean-based (SBM) diet contained 50% (w/w) soybean meal (defatted, protein content 44%) and 11% soy protein isolate; the former is expected to induce intestinal inflammation (16). The product Aquastem™ 300DR (derived from the microalga *Euglena gracilis*) from Kemin, Des Moines, USA was added (2.5%, w/w) to the SBM diet to prepare the β-glucan diet (BG). Likewise, the diets AL and AH were prepared by adding 0.962 and 0.658% (w/w) alginate oligosaccharide, AOS (derived from the macroalga *Laminaria*; Centre d’Etude et de Valorisation des Algues (CEVA), Pleubian, France). The AL diet had a lower overall MW, in particular a higher content of short-chain AOS (over 30% < 3kDa). In addition, AL had 3-10kDa (7%), 10-30kDa (22%) and 30-60kDa (40%) compared to the AH diet that had AOS < 3kDa (3%), 3-10kDa (5%), 10-30kDa (30%) and 30-60kDa (60%). AOS in both AL and AH diets were prepared from the same batch of purified alginates. Hence, they have the same M:G ratio (0.9) and M:G distribution along the polymer (**Supplementary Table 1B**). Thus, BG, AL and AH diets had all the ingredients of the SBM diet in addition to the respective test compound. The experimental fish were fed daily at 5% body weight (offered manually as three rations at 08:00, 13:00 and 18:00) for 30 days. Fish in 5 replicate tanks were allotted to each of the five study groups.

### 2.3 Sampling

At the end of the experimental period, the fish were sacrificed by immersing (5 min) in a lethal dose of 200 mg/l of tricaine methanesulfonate (Argent Chemical Laboratories, Redmond, WA, USA) buffered with an equal amount of sodium bicarbonate. Total length and weight of the individual fish from each treatment group were measured and the information is in **Supplementary Figure 1**. The fish were dissected to collect the posterior intestine (*n* = 5 per group) and snap-frozen in liquid nitrogen. These samples were later stored in a −80°C freezer until further analyses. Blood drawn by tail ablation (18) was collected in a heparinized tube and centrifuged at 5000 g for 10 min at 4°C to collect the plasma (*n* = 5 per group; 5 fish from each tank pooled). Intestine samples (*n*=6-9 per group) were taken to assess the histomorphology.

### 2.4 RNA isolation, mRNA sequencing and bioinformatic analyses

Total RNA was extracted from the frozen intestine samples using Direct-zol™ RNA MiniPrep (Zymoresearch, CA, USA), following the manufacturer’s instructions. The RNA concentration and integrity were determined using Qubit 4 Fluorometer (Thermo Fisher Scientific, Waltham MA, USA) and Tape Station 2200 (Agilent Technologies, Santa Clara, CA, USA). RNA samples exhibiting RIN value ≥ 7 were used for qPCR and preparation of RNA-Seq libraries. Library preparation and sequencing of samples (*n*=5 for each diet group) were done by Novogene Europe (Cambridge, UK). Messenger RNA was purified from total RNA using poly-T oligo-attached magnetic beads. After fragmentation, the first strand cDNA was synthesized using random hexamers followed by the second strand cDNA synthesis. The libraries were end-repaired, A-tailed, adapter ligated, size selected, amplified, and finally purified. The libraries were quantified by Qubit and real-time PCR and size distribution was checked by bioanalyzer. The barcoded libraries were then pooled at equimolar concentrations and loaded on the Illumina NovaSeq 6000 Sequencing system (Illumina, San Diego, CA, USA) to obtain 150 bp paired end reads. For each sample, an average of 22 million filtered reads were obtained with a minimum of 19.8 million reads per sample. The average mapping percentage of the filtered reads was 86% (**Supplementary Table 2**). The bioinformatic analysis of the RNA-Seq data was performed following our previously described protocol (16). In brief, the quality of raw reads was assessed using the FastQC command line, and the tool fastp to filter the reads by considering the Phred quality score (Q ≥ 30). The filtered reads were then aligned to the reference zebrafish genome downloaded from NCBI (release 106) using HISAT2, version 2.2.1 with default parameters. Read counts that belong to each gene were generated using featureCounts version 1.5.3. Differential expression of the genes across the treatment groups was determined by DESeq2 and transcripts with |Log_2_ fold change| ≥ 1 and an adjusted p-value < 0.05 (Benjamini-Hochberg multiple test correction method) were considered significantly differentially expressed. The gene ontology (GO) enrichment analyses were performed using the software Database for Annotation, Visualization and Integrated Discovery version 6.8 with a p value of 0.05 and minimum gene count of 2. The packages ggplot2, pheatmap and GOplot in R were employed to present the data.

### 2.5 qPCR analysis

Genes related to intestinal inflammation, namely *interleukin-1b* (*il1b*), *matrix metalloproteinase-9* (*mmp-9*), *myeloid-specific peroxidase* (*mpx*), *interleukin-10* (*il10*), *chemokine (C-X-C motif) ligand 8a* (*cxcl8a*), *mucin2.1* (*muc2.1*), *mucin5ac* (*muc5ac*) and those of antioxidant enzymes *superoxide dismutase 1* (*sod1*), *glutathione peroxidase 1a (gpx1a), catalase* (*cat*) were selected for qPCR (*n* = 5 for each diet group) and each reaction was done with technical replicates. One µg of total RNA from each sample was reverse transcribed using the QuantiTect reverse transcription kit (Qiagen, Hilden, Germany), according to the manufacturer’s instructions. The cDNA was further diluted 10 times with nuclease-free water and used as a PCR template. PCR reactions were performed using the SYBR green in LightCycler^®^ 96 Real-Time PCR System (Roche Holding AG, Basel, Switzerland) with the following conditions: initial denaturation at 95°C for 10 min, followed by 35 cycles of 95°C for 20 s, 60°C for 30 s and 72°C for 10 s. We designed the primers for the selected genes using the Primer-BLAST tool in NCBI. The primers were then checked for secondary structures such as hairpin, repeats, self and cross dimer by NetPrimer (Premier Biosoft, Palo Alto, USA). The primers for the target genes are listed in **Supplementary Table 3**. Relative expression of selected genes was determined based on the geometric mean of three reference genes (*eef1a1l1*, *rpl13α* and *actb1*). The data were checked for assumptions of normality (Shapiro-Wilk) and homogeneity of variance (Bartlett’s test). Based on the normality and equal variance assumption check results, the statistical difference was determined by Analysis of Variance (ANOVA) or Kruskal-Wallis test. The pair wise comparison between the treatments was done by Tukey’s or Dunn test.

### 2.6 Intestinal histomorphometry

Distal intestine sample were fixed in 3.7% (v/v) phosphate-buffered formaldehyde solution (pH 7.2) at 4°C for 24 h. Standard histological procedures were employed for dehydration, processing, and paraffin embedding as described by Bancroft and Gamble (19). The paraffin blocks thus prepared were sectioned using a microtome (Microm HM3555, MICROM International GmbH, Walldorf, Germany). Four micrometer thick longitudinal sections were cut and mounted on SuperFrost^®^ slides (Menzel, Braunschweig, Germany), and a robot slide stainer (Microm HMS 760×, MICROM International GmbH) was used to stain the slides with Alcian Blue-Periodic Acid Schiff’s reagent (AB-PAS, pH 2.5). First, all acid mucins were stained blue with alcian blue, and in the subsequent PAS reaction, only the neutral mucins were stained magenta. Light microscopy photomicrographs were taken with the Olympus BX61/Camera Color View *III*u (Olympus Europa GmbH, Hamburg, Germany) and the photo program Cell P (Soft Imaging System GmbH, Munster, Germany). The ImageJ software (20) was used for analysing the tissue microarchitecture. To understand the histopathological changes, we measured five parameters of the intestine features: number of eosinophils, goblet cell number, goblet cell size, villi length and width of lamina propria.

### 2.7 Plasma metabolomics

Metabolomic profiling was carried out by MS-Omics (Vedbæk, Denmark). The analysis was carried out using a Thermo Scientific Vanquish LC (Thermo Fisher Scientific, Waltham, U.S.) coupled to Orbitrap Exploris 240 MS (Thermo Fisher Scientific). The company used an electrospray ionization interface as the ionization source. The analysis was performed in positive and negative ionization mode under polarity switching. The ultra-performance liquid chromatography was performed using a slightly modified version of the protocol described by Doneanu et al. (21). Peak areas were extracted using Compound Discoverer 3.2 (Thermo Fisher Scientific). Metabolites in the samples were identified at four levels; Level 1: identification by retention times (compared against in-house standards), accurate mass (with an acceptable deviation of 3 ppm), and MS/MS spectra, Level 2a: identification by retention times (compared against in-house standards), accurate mass (with an acceptable deviation of 3ppm). Level 2b: identification by accurate mass (with an acceptable deviation of 3 ppm), and MS/MS spectra, Level 3: identification by accurate mass alone (with an acceptable deviation of 3 ppm). The obtained metabolomic data were analyzed employing MetaboAnalyst 5.0 (22). The data were log-transformed and auto-scaled (mean-centered and divided by the standard deviation of each variable) before downstream analyses. Principal component analysis was performed using the *mixomics* package in R 4.2.1 to understand the differential clustering of the study groups. Metabolites with a |Log_2_ fold change| ≥ 0.6 and a p value of < 0.05 are reported as significantly altered metabolites. The packages ggplot2 and pheatmap in R 4.2.1 were employed to prepare the illustrations in this article.

## 3 Results

### 3.1 Soybean-based diet altered the expression of key genes related to inflammation

**Figure 1:**
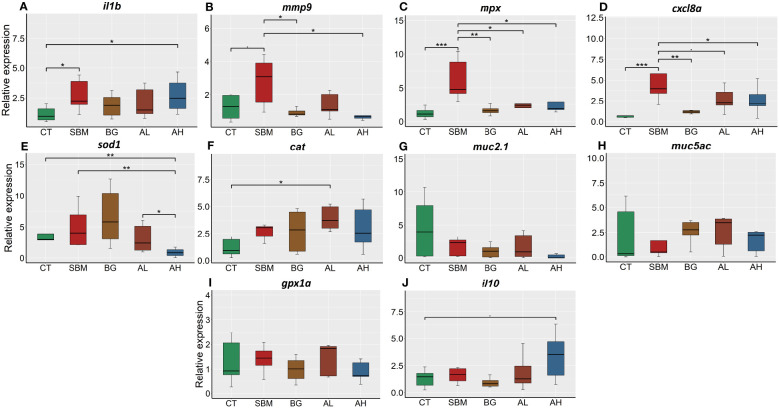
Relative expression of immune genes in the intestine of zebrafish fed different diets. **(A)** *interleukin-1b* (*il1b*); **(B)** *matrix metalloproteinase-9* (*mmp9*); **(C)** *myeloid-specific peroxidase* (*mpx*); **(D)** *chemokine (C-X-C motif) ligand 8a (cxcl8a)*; **(E)** *superoxide dismutase 1* (*sod1*); **(F)** *catalase* (*cat*); **(G)** *mucin2.1* (*muc2.1*); **(H)** *mucin5ac* (*muc5ac*); **(I)** *glutathione peroxidase* (*gpx1a*); **(J)** *interleukin-10* (*il10*). CT- control diet; SBM- soybean diet; BG- algal β-glucan; AL- AOS with 31% < 3kDa; AH- AOS with 3% < 3kDa. Asterisk * *p* < 0.05, ** *p* < 0.01 and *** *p* < 0.001, • *p* < 0.1. Each treatment group consisted of five biological replicates.

To gather evidence on soybean meal-induced inflammatory response in zebrafish, we examined the relative expression of selected inflammatory genes in the intestine of the fish fed a soybean meal-based diet for 30 days. The relative expression of *il1b* was significantly (p < 0.05) increased in the SBM and AH groups compared to CT group (**Figure 1A**). Furthermore, the SBM group had significantly higher expression (p < 0.05) of *mmp9* compared to the BG and AH groups (**Figure 1B**) and significantly (p < 0.001) higher expression of *mpx* compared to the CT group (**Figure 1C**). However, the expression of *mpx* in the BG, AL and AH groups was significantly lower compared to the SBM group. The expression of *cxcl8a* was significantly higher in the SBM group (p < 0.001) compared to CT, BG and AH groups (**Figure 1D**). In addition, we observed significant differences in the expression of the antioxidant genes *sod1* and *cat* (**Figures 1E, F**); the expression of *sod1* was significantly lower in the AH group compared to the CT, SBM and AL groups and the expression of *cat* was significantly higher in the AL group compared to the CT group. We did not detect any statistically significant differences in the expression of the mucin genes, *muc2.1* and *muc5ac*, the gene of the antioxidant enzyme, *gpx1a*, and the anti-inflammatory gene, *il10* (**Figures 1G–J**).

### 3.2 Intestinal transcriptome profile reflected soybean-induced inflammation

**Figure 2:**
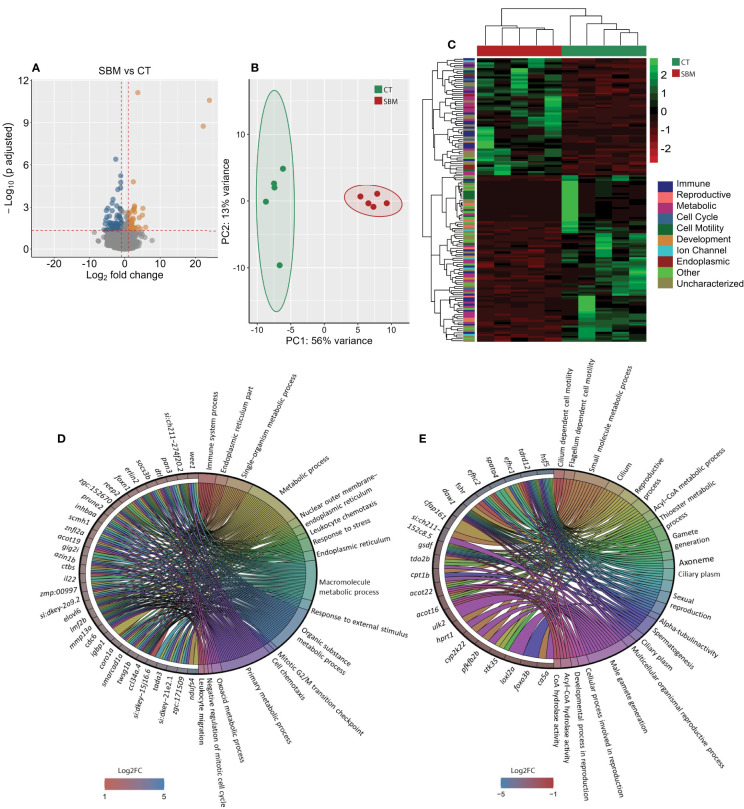
Transcriptome-based differences in the intestine of zebrafish from the soybean group compared to the control group. Volcano plot **(A)**, PCA plot **(B)** and heatmap **(C)** of the differentially expressed genes in the soybean (SBM) group compared to the control (CT) group. Chord diagram showing the link between the enriched GO terms in the soybean group and the associated genes that were upregulated **(D)** and downregulated **(E)** in the soybean (SBM) group compared to the control (CT) group. The enriched GO terms are colour-coded and on the right side of the circles one finds the differentially expressed genes contributing to the enriched GO terms that are shown on the left of the circles. The gradient colour bar intensity varies with the Log2 fold change (adjusted p-value < 0.05 and |Log2 fold change| ≥ 1). There are five biological replicates in each study group.

To gain a deeper understanding of the effects of soybean meal-induced inflammation, we analyzed the intestinal transcriptome of zebrafish fed the soybean meal-based diet. A comparison of the SBM group with the CT group revealed 141 differentially expressed genes (DEGs), of which 58 were upregulated and 83 were downregulated in the SBM group (**Figure 2A** and **Supplementary Table 4**). The principal component analysis (PCA) plot shows the differential clustering of the SBM and CT groups along the first principal component (PC1), which explains 56% variability in the data (**Figure 2B**). Hierarchical clustering (**Figure 2C**) revealed a clear separation of upregulated and downregulated DEGs in the SBM group compared to the CT group.

The GO enrichment analysis based on the upregulated DEGs revealed significant enrichment of several GO terms including those linked to immune system process, endoplasmic reticulum (ER) part, leukocyte chemotaxis, response to stress, response to external stimulus and leukocyte migration (**Figure 2D**). GO enrichment analysis employing the downregulated DEGs revealed terms like cilium dependent motility, flagellum dependent cell motility, sexual reproduction, alpha-tubulin activity and male gamete generation (**Figure 2E**).

### 3.3 Algal β-glucan targets distinct immune-related genes

**Figure 3:**
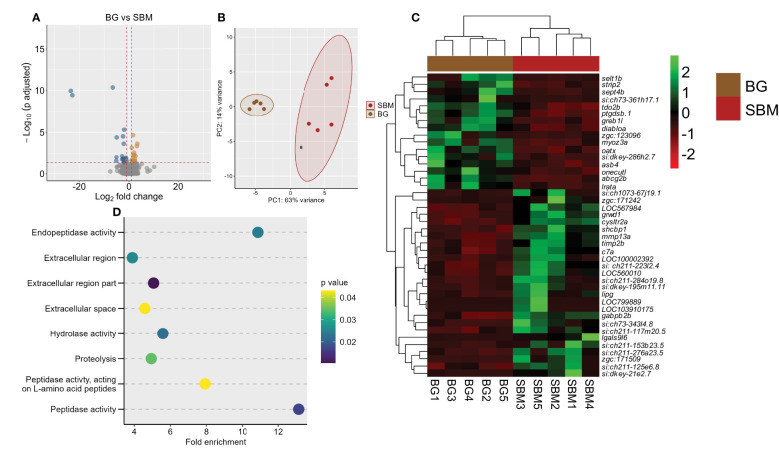
Transcriptome-based differences in the intestine of zebrafish from the algal β-glucan group compared to the soybean group. Volcano plot **(A)**, PCA plot **(B)** and heatmap **(C)** of the differentially expressed genes in the algal β-glucan (BG) group compared to the soybean (SBM) group. Transcripts with an adjusted p-value below 0.05 and |Log_2_ fold change| ≥ 1 were considered as significantly differentially expressed. Dot plot **(D)** showing enriched GO terms in the BG group based on the genes that were differentially downregulated compared to the SBM group. The gradient colour bar intensity of GO terms varies with the p value of each GO term. There are five biological replicates in each study group.

We performed a comparison of the transcriptome of the BG and SBM groups. Forty-two genes were differentially expressed, of which 16 were upregulated and 26 were downregulated in the BG group (**Figure 3A** and **Supplementary Table 5**). The PCA plot shows the differential clustering of the SBM and BG groups along the PC1, which reveals 63% variability in the data (**Figure 3B**). Hierarchical clustering (**Figure 3C**) revealed a clear separation of DEGs in the SBM group compared to the BG group. Several downregulated DEGs were immune-related, for example *GTPase IMAP family member-like gimap7* (*LOC799889*)*, gimap8* (*LOC103910175*), *lectin, galactoside-binding, soluble, 9 (galectin 9)-like 6* (*lgals9l6*), *matrix metalloproteinase-13a* (*mmp13a*)*, chemokine ccl-c17a* (*LOC100002392*), *TIMP metallopeptidase inhibitor 2b* (*timp2b*) and *complement component* (*c7a*). The GO enrichment analysis employing the downregulated DEGs revealed the enrichment of terms like endopeptidase activity, hydrolase activity and proteolysis (**Figure 3D**). However, the upregulated DEGs did not reveal any significant GO enrichment.

### 3.4 Specific shift in the expression of immune-related genes caused by AOS

**Figure 4:**
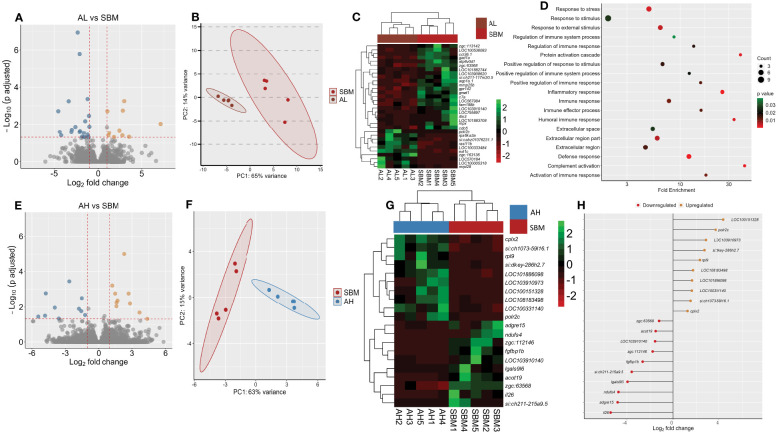
Transcriptome-based differences in the intestine of zebrafish fed AOS with 31% <3kDa and AOS with 3% <3kDa compared to the soybean group. Volcano plot **(A)**, PCA plot **(B)** and heatmap **(C)** of the differentially expressed genes in the AL (AOS with 31% <3kDa) group compared to the soybean (SBM) group. Dot plot **(D)** showing the enriched GO terms in the AL group based on the differentially downregulated genes compared to the SBM group. The gradient colour bar intensity of GO terms varies with the p value of each GO term. Volcano plot **(E)**, PCA plot **(F)**, heatmap **(G)** and differentially expressed genes **(H)** obtained by comparing the AH (AOS with 3% < 3kDa) group with the soybean (SBM) group. Transcripts with an adjusted p value below 0.05 and |Log_2_ fold change| ≥ 1 were considered as significantly differentially expressed. There are five biological replicates in each study group.

We first compared the intestine transcriptome of the AL group with the SBM group. The analysis revealed 32 DEGs, of which 10 were upregulated and 22 were downregulated in the AL group (**Figure 4A**). The PCA plot shows the differential clustering of the SBM and AL groups along the PC1, which explains 65% of variability in the data (**Figure 4B**). Hierarchical clustering (**Figure 4C**) revealed a clear separation of differentially expressed genes in the SBM group compared to the AL group. Several downregulated DEGs were immune related; *chemokine (C-C motif) ligand 36, duplicate 1* (*ccl36.1*), *intelectin 3* (*itln3*), *CD59A glycoprotein-like* (*LOC103910140*), *aquaporin 1a* (*aqp1a.1*), *NLR family CARD domain-containing protein 3-like* (*LOC101882744*), *myeloid-specific peroxidase* (*mpx*), *c7a*, *G protein-coupled receptor* 142 (*gpr142*) and *matrix metalloproteinase-25b* (*mmp25b*) (**Figure 4C** and **Supplementary Table 6**). GO enrichment analysis employing the downregulated DEGs revealed terms like response to stress, inflammatory response, immune response, humoral immune response and complement activation (**Figure 4D**). The upregulated DEGs based analyses revealed the enrichment of intracellular organelle lumen (**Supplementary Table 7**).

Comparison of the AH group with the SBM group revealed 20 DEGs of which 10 were upregulated and 10 were downregulated in the AH group (**Figure 4E** and **Supplementary Table 8**). The PCA plot shows the clustering of samples into SBM and AH groups. The clusters are separated from each other along the PC1, which explains 63% of variability in the data (**Figure 4F**). Hierarchical clustering (**Figure 4G**) revealed a clear separation of up- and downregulated DEGs in the AH group compared to the SBM group. The downregulated DEGs-based GO analysis revealed enrichment of negative regulation of immune system processes (**Supplementary Table 9**). Our analysis did not detect a significant GO term enrichment based on the upregulated DEGs. The upregulated DEGs were immune related: *B-cell receptor CD22 (LOC100151328), NLR family CARD domain-containing protein 3-like (LOC108183498), Fc receptor-like protein 5 (LOC101886098)* and *macrophage mannose receptor 1-like (LOC100331140).* The downregulated DEGs were, among others, *interleukin 26* (*il26)*, *adhesion G protein-coupled receptor E15* (*adgre15*), *lectin galactoside-binding, soluble, 9 (galectin 9)-like 6 (lgals9l6)* and *CD59 glycoprotein-like (LOC103910140)* (**Figure 4H**).

**Figure 5:**
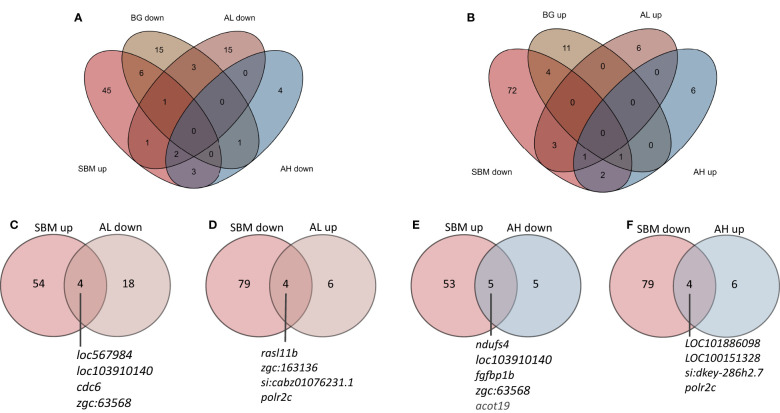
Venn diagrams showing the total number of genes that were altered by the experimental diets. The genes that were differentially upregulated in the SBM vs CT comparison **(A)** but downregulated in the BG, AL and AH vs SBM comparisons. The genes that were differentially downregulated in the SBM vs CT comparison **(B)** but upregulated in the BG, AL and AH vs SBM comparisons. Genes that were altered in the SBM group had mRNA levels in the AL **(C, D)** and AH **(E, F)** groups, respectively, similar to that in the control group.

To find out if the AOS has the capacity to shift the expression of genes that were altered by SBM, we examined the common DEGs from the transcriptome comparisons, viz. SBM vs. CT as well as AL vs SBM and AH vs SBM (**Supplementary Figure 2** and **Figures 5A, B**). Here we list the DEGs that had contrasting expression patterns in the abovementioned comparisons. The expression of four upregulated DEGs in SBM vs CT comparison, namely *fin TRIM family*, *member 37* (*LOC567984*), *CD59A glycoprotein-like* (*LOC103910140*), *cell division cycle 6 homolog* (*cdc6*), *alcohol dehydrogenase 5-like* (*zgc:63568*) and four downregulated DEGs in SBM vs CT comparison namely *RAS like family 11 member B* (*rasl11b*), *HECT and RLD domain containing E3 ubiquitin protein ligase 56.3* (*zgc:163136*), *si:cabz01076231.1*, *RNA polymerase II subunit* C (*polr2c*) were shifted in the AL group to a level almost similar to that in the control group (**Supplementary Figure 2** and **Figures 5C, D**). On the other hand, the expression of five upregulated DEGs in SBM vs CT namely *NADH:ubiquinone oxidoreductase subunit S4* (*ndufs4*), *CD59A glycoprotein-like* (*LOC103910140*), *fibroblast growth factor binding protein 1b* (*fgfbp1b*), *alcohol dehydrogenase 5-like* (*zgc:63568*), *acyl-CoA thioesterase 19* (*acot19*) and four downregulated DEGs in SBM vs CT comparison namely *Fc receptor-like protein 5* (*LOC101886098*), *B-cell receptor CD22* (*LOC100151328*), *si:dkey-286h2.7*, *RNA polymerase II subunit* C (*polr2c*) were shifted in the AH group to levels almost similar to those in the control group (**Figures 5E, F**). Venn diagram reveals the two common downregulated DEGs (*LOC103910140*, *zgc:63568*) (**Figures 5C, E**) and one common upregulated (*polr2c*) (**Figures 5D, F**) DEG in the AL and AH diet groups compared to the SBM group, respectively.

A direct comparison of AL versus AH group revealed 14 DEGs, of which 2 were upregulated and 12 were downregulated in the AL group. Among the downregulated DEGs, immune genes like *ccl36.1* had a 12-fold and *C-reactive protein -6* (*crp6*) had a 5-fold downregulation in the AL group compared to the AH group (**Supplementary Table 10**). Furthermore, these 2 DEGs caused the enrichment of the GO term “response to virus” (**Supplementary Table 11**).

### 3.5 Soybean-based diets (both with and without glucans or AOS) altered the plasma metabolome

**Figure 6:**
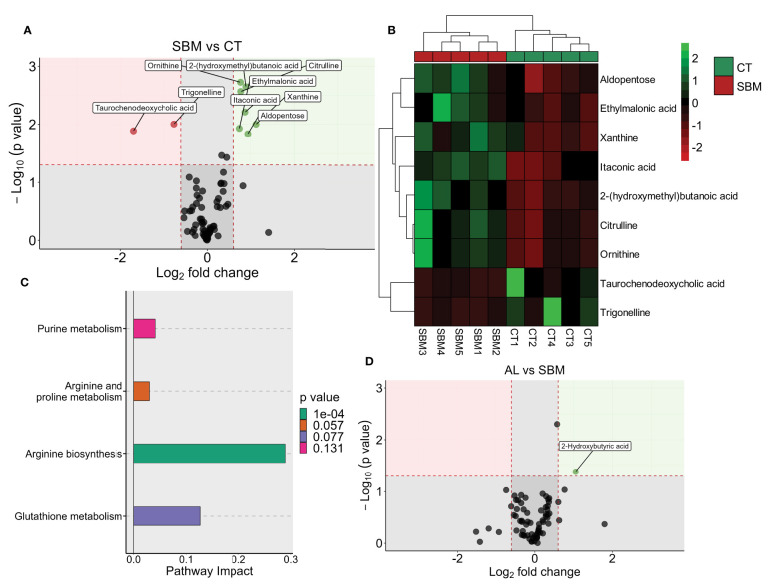
Diet-induced changes in plasma metabolites of zebrafish. Volcano plot **(A)**, heatmap **(B)** and enriched KEGG pathways **(C)** by the differentially altered metabolites in the soybean (SBM) group compared to the control (CT) group (|Log_2_ fold-change| ≥ 0.6, p value < 0.05). Volcano plot **(D)** showing the differentially abundant metabolites in the AL group (AOS diet with 31% < 3kDa) compared to the soybean (SBM) group. There are five biological replicates in each study group.

To gain deeper insights into the impact of different dietary treatments, we compared the plasma metabolome of the various treatment groups. We identified a total of 71 metabolites (level 1). Partial least squares discriminant analysis revealed a group-based clustering of the samples (**Supplementary Figure 3**). Comparison of the SBM group with the CT group revealed aldopentose, ethylmalonic acid, xanthine, itaconic acid, 2-(hydroxymethyl) butanoic acid, citrulline, ornithine, taurochenodeoxycholic acid and trigonelline as the significantly altered metabolites (**Figures 6A, B**; **Supplementary Figure 4** and **Supplementary Table 12**). The pathway analysis using these nine significantly altered metabolites identified arginine biosynthesis as the significantly enriched pathway (**Figure 6C**). Comparison of the BG group with the SBM group revealed pantothenic acid and isocitric acid as the significantly altered metabolites (**Supplementary Figure 5A** and **Supplementary Table 13**). Furthermore, the AL group versus the SBM group revealed 2-hydroxybutyric acid as the significantly abundant metabolite (**Figure 6D**; **Supplementary Figure 5B** and **Supplementary Table 14**). We did not find any significantly altered metabolite from the AH vs SBM comparison.

### 3.6 AOS altered the intestinal histomorphology

**Figure 7:**
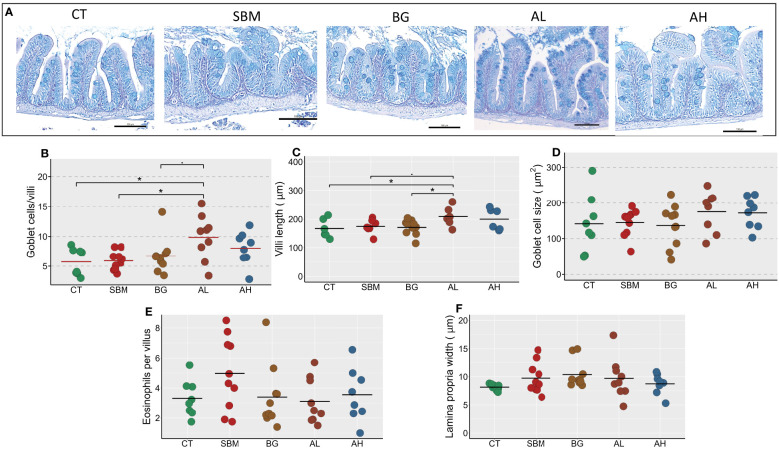
Diet-induced histomorphological changes in the intestine of zebrafish. Representative histological images **(A)** showing the changes in tissue architecture of the intestine of zebrafish stained with AB-PAS. Scale bar = 100 μm. The measured parameters include **(B)** Goblet cells per villus **(C)** Villi length **(D)** Goblet cell size **(E)** Eosinophils per villus **(F)** Lamina propria width. Horizontal bars indicate mean values. * indicates p < 0.05 and *p* < 0.1 (n = 6-9 per group). CT- control diet; SBM- soybean diet; BG- algal β-glucan diet; AL- AOS diet with 31% < 3kDa; AH- AOS diet with 3% < 3kDa.

We investigated histological changes in the intestine of zebrafish to understand the effect of different diets (**Figure 7A**). We found a significantly higher number of goblet cells per villi (p < 0.05) in the AL group compared to the CT and SBM groups (**Figure 7B**). We also found an increase in the villi length in the AL group compared to the CT and BG groups (**Figure 7C**). The diets seem to have no effect on goblet cell size, eosinophils and lamina propria width in zebrafish (**Figures 7D–F**).

## 4 Discussion

Prebiotics are often administered through diet to obtain a “synergistic or complementary synbiotic” effect, and currently, scientists are gathering evidence on the IBD-alleviating potential of this approach. The belief is that dietary prebiotics change the composition of intestinal microbiota, which influences mucosal as well as systemic immune responses in a host (23). In one of our previous studies (13), we profiled the intestinal bacterial communities in Atlantic salmon fed two levels of AOS (0.5 and 2.5%). We reported the potential ability of 0.5% AOS to stimulate the proliferation of bacteria with SCFA-producing capacity. The same product was added to the AL diet of the current study. For comparative purposes, we formulated the AH diet that contained an AOS with a lower proportion of < 3kDa. The two products were incorporated at 0.962% (AL) and 0.658% (AH) (both w/w) into the diet of zebrafish, taking into consideration the content of the active component. We performed an *in vivo* study to compare the anti-inflammatory effects imparted by two AOS products (with 31% < 3kDa and with 3% < 3kDa), using an intestine inflammation model in adult zebrafish. We targeted the transcriptome and metabolome of the fish to evaluate the anti-inflammatory potential of AOS. We have also studied the transcriptome and metabolome of zebrafish fed an algal β-glucan that we studied previously (16).

The generated transcriptomic and metabolomic profiles revealed the distinct responses evoked by the products (based on the comparison with the SBM group). Downregulated DEGs-based enriched GO terms of the AL group were complement activation, inflammatory response and humoral response, compared to the negative regulation of the immune system in the case of the AH group. The significantly abundant plasma metabolite in the AL group was 2-hydroxybutyric acid. Histological evaluation indicated that the AL group had more goblet cells and longer intestinal villi.

### 4.1 Dietary soybean meal altered the expression of genes linked to inflammation, endoplasmic reticulum, reproduction and cell motility

Soybean meal contains several anti-nutritional factors (ANFs) including saponins, lectins, isoflavones, and β-conglycinin (24). These ANFs can hamper growth, reduce digestive enzyme activity, and alter the gut mucosal integrity to induce inflammation. Such an inflammatory response could be due to soy saponins as reported in fish studies (25, 26). Increased granulocyte recruitment and higher expression of inflammatory marker genes (*il1b* and *il8*) were the characteristics described in zebrafish larvae fed a diet containing soybean meal and soy saponin (27). In our previous studies, we found that the dietary soybean meal (50% inclusion) affected barrier-related genes in the intestine of juvenile zebrafish (28) and soy saponin developed inflammation features such as increased lamina propria width, infiltration of immune cells, and increased expression of genes related to antimicrobial peptides and ion transport in the intestine of Atlantic salmon (26). In the present study, the expression of inflammatory marker genes (*il1b*, *mpx*, *cxcl8a*) were upregulated in the SBM group. The proinflammatory cytokine IL-1β is secreted by innate immune cells and is an important mediator of inflammatory response (29). The chemokine CXCL8A is a neutrophil chemoattractant that stimulates the migration of neutrophils from blood to the inflamed sites. Granulocytes (mainly neutrophils) are the first responders that migrate to an inflamed site and a high concentration of granulocytes represents a transition from an acute phase to a chronic inflammatory state (30). We also gathered evidence on increased presence of neutrophils in the intestine of larval zebrafish (16) and in the present study the expression of the neutrophil marker *mpx* was elevated by the SBM diet. Moreover, MPX was found to be involved in the production of ROS in the mucosa of patients suffering from intestinal inflammation (31). The MMPs secreted by neutrophils degrade the extracellular matrix, facilitating the transendothelial migration of neutrophils to the inflamed sites (32). In the present study, several inflammation-related GO terms like leukocyte chemotaxis, leukocyte migration were enriched in the SBM group with significantly upregulated immune genes (*mmp13a*, *coro1a*, *il22, ccl34a.4*, *cd59*, *foxn*, *gig2i*). The metalloprotease gene *mmp13* codes for an endopeptidase that plays a critical role in intestinal epithelial barrier disruption and is therefore considered a potential therapeutic agent for treating IBD (33). LPS-induced goblet cell depletion, ER stress, permeability and tight junction alterations were reduced in the gut of *Mmp13* knockout mice (33). The gene *il22* codes for a cytokine that regulates the intestinal barrier integrity and its expression was altered during inflammation (34). A previous study on juvenile Jian carp (*Cyprinus carpio* var. Jian) reported that soybean β-conglycinin can also cause intestinal damage and induce inflammation and oxidative stress as a result of the elevated expression of inflammatory cytokine *il-8*, *tumor necrosis factor-α* (*tnf-α*), and *transforming growth factor-β* (*tgf-β*) genes and reduction of the anti-oxidant enzymes SOD and CAT (35). Hence, the negative effects of soybean meal can be compounded by the actions of all the antinutritional factors. For instance, soybean lectins can potentiate the detrimental effects of saponin on epithelial barrier function (36). Furthermore, dietary soybean meal can also have other metabolic effects like altering the cholesterol metabolism and hampering reproductive development (37, 38). In the present study, several downregulated DEGs in the SBM group significantly enriched the GO term sexual reproduction. These results corroborated those reported in our previous article; 50% soybean meal feeding altered genes related to reproduction and cholesterol metabolism in zebrafish (16, 28). This could be attributed to isoflavones present in soybean meal, which can bind to oestrogen receptors (39). As reported in previous studies, alteration in the membrane cholesterol by soy saponin might have affected cell motility and lipid metabolism by influencing the functioning of ER (40, 41). Furthermore, dietary soybean meal can increase the rate of respiration (16), thereby increasing the production of reactive oxygen species (42) and aggravating the inflammatory response (43). Soybean meal diet increased the oxygen consumption (16) and altered the genes related to oxidoreductase activity in zebrafish (28). Thus, the intestinal inflammatory response to soybean meal can be a direct effect of anti-nutritional factors or due to cumulative metabolic changes caused by multiple factors in the soybean diet.

### 4.2 Distinct changes in the intestine of adult zebrafish fed soybean meal and algal β-glucan or AOS

Defects in the barrier function caused by intestinal structural changes can increase luminal antigen penetration and the associated chemokine-induced recruitment of neutrophils. We found that the expression of genes associated with neutrophil recruitment (*mpx* and *cxcl8a*) and barrier disruption (*mmp9*) was downregulated in the AOS and BG fed groups. The expression of proinflammatory cytokine *il1b* was upregulated in the SBM and AH group. Conversely, the expression of *il1b* was not altered in the AL group compared to the control group suggesting an immune modulation in the zebrafish intestine by the AL diet. Furthermore, in the AL group the downregulated DEGs (*cd59*, *c7a, mpx*, *ccl36.1*, *itln3*, *aqp1a.1*, *nlrc3*, *gpr142 and mmp25b*) enriched the GO terms inflammatory response, complement activation and humoral immune response. Note that *mpx* and *mmp* (*mmp25b*) were downregulated in the AL group. Furthermore, the expression of the gene, *cat* was upregulated in the AL group, and this antioxidant is a key regulator of ROS generated during inflammatory conditions (44). It should be noted that catalase activity was lower in patients suffering from intestinal inflammation (45) and catalase administration can reduce ROS levels and ameliorate inflammation, as shown in colitis mice models (44). Intestinal epithelial cells are sources of complement components and appropriate regulation of complement activation is essential to prevent intestinal epithelial cell damage. Increased complement activation has been associated with the pathogenesis of IBD (46). The C7A protein is part of the membrane attack complex (MAC), and the downregulation of gene expression of this component points to the prevention of complement activation. Therefore, the suppression of several processes related to inflammation by the downregulated DEGs and the increase in the antioxidant gene *cat* in the AL group suggest the ability of AOS (AL) to reduce the intestinal inflammation induced by the dietary soybean.

Conversely, in the AH group, we found one GO term, viz. negative regulation of immune system process, enriched by the downregulated DEGs (*lgals9l6*, *CD59 glycoprotein-like*). The downregulated DEG in the AH group, *lgals9l6* that codes for protein galactoside-binding, soluble, 9 (galectin 9)-like 6, is an ortholog of human *LGALS9* (galectin 9/Gal-9). Gal-9, a β-galactoside binding lectin with a carbohydrate recognition domain, is expressed in human crypt cells and its expression is lowered in IBD patients (47). Furthermore, mice lacking *gal-9* were reported to have impaired intestinal mucosal antigen-specific IgA response and were more susceptible to developing watery diarrhoea (47). Because CD59 prevents the activation of the complement system and the associated assembly of MAC, the decrease in epithelial expression of CD59 in IBD patients renders the epithelial cells prone to complement lysis and may lead to destruction of gut epithelium (48). Furthermore, the comparison of AH group with the SBM group revealed the upregulation of several immune genes (*il26*, *cd22*, *nlrc3*, *cd206*). The gene *il26* is a mediator of inflammation and is overexpressed in activated or transformed T cells (49). The protein CD22 is abundantly expressed on the cell surface of activated B-lymphocytes and it can negatively regulate lamina propria eosinophil levels, as in the case of mice (50). Based on these facts, we speculate that the AL group is effective in reducing inflammation.

Venn diagrams created to understand the differential effects of AL and AH on the expression of genes in zebrafish intestine revealed that the results of the AL vs SBM comparison was distinct from those of AH vs SBM comparison. We found only three common DEGs (*loc103910140*, *zgc:63568*, *polr2c*) in the two comparisons; two (zgc:63568 and *loc103910140*/*CD59*) of these were downregulated DEGs and one was an upregulated (*polr2c*) DEG. As mentioned before, the protein CD59 prevents the complement activation and MAC formation. We performed a direct transcriptomic comparison of the AL group with AH group to delineate further the specific effects of the two products. This comparison revealed the downregulation of *ccl36.1* and *crp6* in the AL group. The zebrafish gene *crp6* is an ortholog of human *CRP*, which is used as a biomarker of systemic inflammation and has been reported as a valuable marker of IBD (51). In our previous study, we have reported that the positive effects of yeast-derived β-glucan on soybean meal-induced inflammation could also be due to a downregulation in the expression of *ccl36.1* (28). In the present study, dietary algal β-glucan downregulated the DEGs (*zgc:171509, timp2b, lipg, mmp13a, c7a*) linked to the GO terms like endopeptidase activity and proteolysis. We found that the immunostimulant can suppress the expression of *mmp13a* while the expression of *mmp13* was upregulated in Atlantic salmon infested with sea lice in response to chronic tissue damage (52). The genes *mmp13* and *timp2* have an essential role during tissue remodelling because the expression of the molecules determine the intricate extracellular matrix turnover (53, 54). Therefore, these genes could be markers of tissue damage caused by the dietary soybean. Furthermore, as noted in this study on adult fish, in larval zebrafish also algal β-glucan reduced endopeptidase and proteolytic activity (16).

### 4.3 AOS diet altered the histological architecture of the intestine

We studied five histomorphological parameters of the intestine and found that the AL group had longer villi with significantly higher number of goblet cells. Goblet cells are responsible for the synthesis, storage, and release of intestinal mucin proteins. Mucus production is an indication of a healthy barrier function as it restricts the entry of pathogens and unwanted luminal antigens into the intestine. It is reported that oligosaccharides can support the mucosal barrier function by stimulating intestinal goblet cells to produce more mucus (55). More mucus cells per villi is an indication that more intestinal cells differentiate into goblet cells to reinforce the barrier. We noted changes specific to the AL group. AL diet fed fish had longer villi and more goblet cells. More goblet cells, longer villi and an increase in the villus height-to-crypt depth (V:C) ratio were reported in a study on AOS diet fed pigs that had better growth (11). β-glucans increased the V:C ratio as well as the average body weight of broiler chicken (56). Mannan oligosaccharide enhanced the growth, increased the villi height and decreased the intestine crypt depth of the juvenile striped catfish (*Pangasianodon hypophthalmus*) (57). There are not many reports on AOS induced alteration in V:C ratio and its correlation with the growth of fishes. Since zebrafish lacks intestinal crypts (58), we cannot relate the growth to the V:C ratio. Nevertheless, increased villus height has been associated with increased nutrient absorption, higher transport of nutrients and improved growth in mammals and fish (59, 60). However, the AL diet did not stimulate the growth of zebrafish. Our previous study on the larval zebrafish model also did not reveal any effect of the SBM and β-glucan diets (also used in the present study) on the standard length (16). Conversely, SBM diet caused several developmental defects like impairment of eye, swim bladder and skeletal deformities in the larval zebrafish (16). Zebrafish is known to have a determinate growth (46) and therefore fish of age 20–40 dpf is considered suitable for a reliable growth study. During this period, energy is predominantly allocated for rapid growth and this time window permits a 40-fold increase in body weight (61). However, in our study we did not find any changes in the growth of the AL group as the feeding experiment was conducted using adult zebrafish. A previous study has also reported that inclusion of soybean meal can stimulate an inflammatory response in the intestine without any effect on the growth of zebrafish (62).

### 4.4 Plasma metabolites indicate soybean meal-induced inflammation, and AOS and algal β-glucan induced SCFA and vitamin production

To our knowledge, this is the first study on the metabolites of zebrafish plasma. Plasma metabolomics can give indications of the systemic perturbations caused by intestinal inflammation (39). Only few metabolites have been detected in plasma from zebrafish due to the small sample amount that can be retrieved from the fish. A comparison of the SBM group with the control group yielded 9 differentially abundant metabolites, out of the 71 detected metabolites. Among the altered metabolites, itaconic acid was significantly abundant in the SBM group compared to the control group. Itaconic acid is considered a biomarker of inflammation, and M1 macrophages are known to produce substantial amounts of itaconate (63). Furthermore, itaconate concentration was markedly increased during lipopolysaccharide- and interferon-γ-induced activation of mammalian macrophages (64), probably due to polarization of macrophages to their M1 phenotype. We also found a decrease in the metabolite taurochenodeoxycholic acid (TCDCA) in the SBM fed group. TCDCA, the secondary bile acid that is conjugated with taurine, is a derivative of the primary bile acid chenodeoxycholic acid. Secondary bile acids are microbiota-associated metabolites and studies have reported an increase in primary bile acids and a reduction in secondary bile acids in IBD patients (65). Furthermore, the amino acid residues in soybean protein have a high bile acid-binding ability and can suppress enterohepatic circulation, even in fishes (66). Therefore, the decreased concentration of TCDCA in plasma is also likely due to soybean feeding. On the other hand, arginine nitric oxide and arginine urea pathways are implicated in the pathogenesis of IBD; in the former case NOS2 (the inducible form of nitric oxide synthase [iNOS]) metabolizes L-arginine to NO and L-citrulline and in the latter arginases (ARG1 and ARG2) catalyse the conversion of arginine to urea and ornithine. The abundance of ornithine and citrulline was higher in the plasma of zebrafish fed the inflammation-inducing diet, as reported for IBD patients (67). However, pathway analysis using metabolites with significantly higher abundance in SBM group detected arginine biosynthesis as the significantly enriched pathway. Our results point to the enrichment of the arginine biosynthesis pathway and upregulation of ornithine and citrulline. Increase of arginine biosynthesis in the SBM group could be a compensatory response in the body due to decreased arginine availability associated with the inflammatory response (68).

SCFAs are produced by microbial fermentation of non-digestible carbohydrates in the posterior segment of the intestine of mammals (69). We found significantly higher levels of 2-hydroxybutyric acid (2-HB) in the plasma of the AL group. While a high fat diet decreased the abundance of 2-HB in the serum of mice, dietary polysaccharide increased the metabolite (70). The same study reported that pre-treatment of macrophages with 2-HB can significantly decrease LPS-induced up-regulation of TNF-α. In the present study, proinflammatory genes were downregulated in the AL group with a high abundance of 2-HB. Interestingly, we found that AOS (AL) can elevate 2-HB, which was positively correlated with *Alloprevotella* in another study (71). Furthermore, dietary AOS could increase the abundance of *Alloprevotella* and butyric acid which are positively correlated (72). The AL and AH groups exhibited distinct transcriptomic and metabolomic responses although the two diets differed only in terms of the percentage of the low molecular weight fraction (AL, with 31% < 3kDa and AH, with 3% < 3kDa). Our results indicate that this difference can affect the immune modulatory and prebiotic potential of the diet; the AL diet was more effective in reducing the intestinal inflammation compared to the AH diet. Low molecular weight polysaccharides are more soluble and have greater fermentability (73). Low and high molecular polysaccharides are utilized by different intestine bacteria (74), and the former type is fermented faster to produce SCFAs and have better prebiotic potential (75). This could be the reason for the detection of a SCFA in the plasma of the AL group.

A comparison of the BG group with the SBM group indicated an increase in the abundance of pantothenic acid also known as vitamin B5 (VB5). A previous study has found an inverse correlation between dietary VB5 intake and serum CRP concentration (marker of inflammation) in humans (76). Although we observed a downregulation of *crp6* in the AL group compared to the AH group, such changes were not noted for the BG group. A study on mice revealed that VB5 could enhance the phagocytosis of macrophages to reduce the pathogen load in macrophages (77). In addition, *in vitro* studies have shown that VB5 can increase glutathione levels in cells, suggesting a role of VB5 as an antioxidant to reduce cell damage (78). Although previous studies have not indicated a connection between pantothenic acid and dietary β-glucan, it is possible that algal β-glucan might have stimulated the proliferation of gut microbes such as *Bacteroides fragilis*, *Prevotella copri* and *Ruminococcu*s spp. that possess the genes to synthesize vitamin B5 (79).

## 5 Conclusion

Dietary soybean affected both the expression of inflammatory marker genes (*il1b, mpx*, *cxcl8a*) and metabolites like itaconic acid and taurochenodeoxycholic acid in the intestine of zebrafish. Conversely dietary AOS with a higher percentage of low molecular weight reduced the expression of several inflammatory marker genes, increased goblet cell number, villi height and a SCFA in the plasma. The BG diet suppressed several immune genes linked to the endopeptidase activity and proteolysis, suggesting a possible role of algal β-glucan in controlling the tissue damage caused by dietary soybean. In the future, it would be interesting to study the impact of structurally different AOS on the microbiota composition and SCFAs in zebrafish and explore the synergetic effect of AOS and algal β-glucan in reducing soybean induced intestinal inflammation.

## Data availability statement

The datasets presented in this study can be found in online repositories. The names of the repository/repositories and accession number(s) can be found here: SRA Bioproject, PRJNA896758.

## Ethics statement

The approval for the conduct of this study was obtained from the Norwegian Animal Research Authority, FDU (Forsøksdyrutvalget ID-22992). We adhered to the rules and regulations regarding the research on experimental animals; FOR-2015-06-18-761. Also, the biosafety rules and regulations stipulated by the Health, Safety and Environment system of the Faculty of Biosciences and Aquaculture, Nord University, were followed during the experiment.

## Author contributions

SR, VK, and JD designed the study. RP provided AOS products, KM supplied the algal β-glucan and helped to plan the study. JD formulated and prepared the experimental feeds. SR and AG performed the feeding experiment. AG, SR, and YA did the sampling. SR did the molecular analyses. AG and SR performed the bioinformatic analysis. SR and VK wrote the manuscript. All authors contributed to the article and approved the submitted version.
