# Supplementary material for: Potential of algae-derived alginate oligosaccharides and β-glucan to counter inflammation in adult zebrafish intestine
Source: Front Immunol. 2023 May 19;14:1183701. doi: 10.3389/fimmu.2023.1183701 (PMC10235609; doi:10.3389/fimmu.2023.1183701)
Supplement: Supplementary file 2 [file Presentation_1.pptx]

## Slide 1
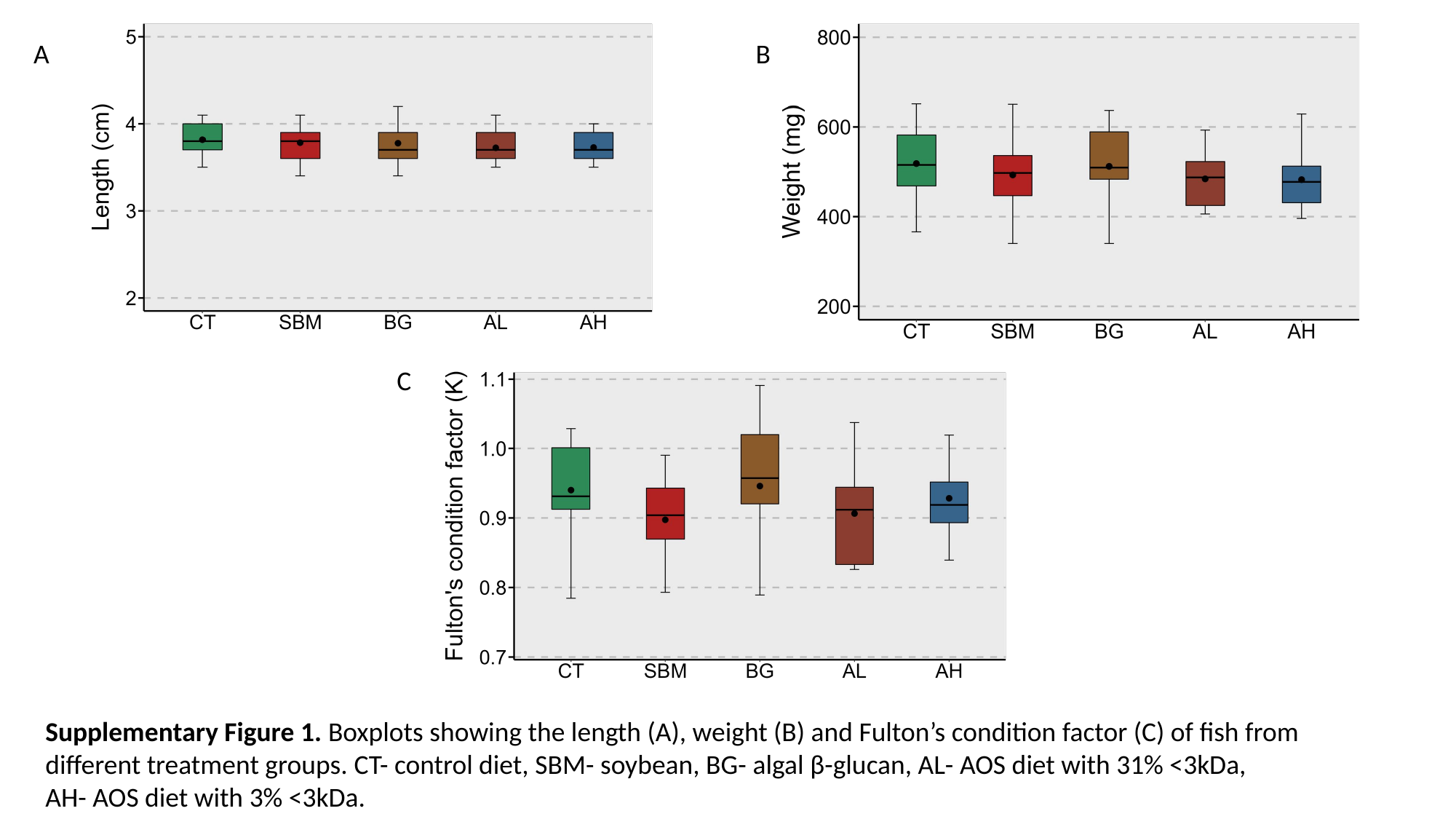

A
B
C
Supplementary Figure 1. Boxplots showing the length (A), weight (B) and Fulton’s condition factor (C) of fish from
different treatment groups. CT- control diet, SBM- soybean, BG- algal β-glucan, AL- AOS diet with 31% <3kDa,
AH- AOS diet with 3% <3kDa.

## Slide 2
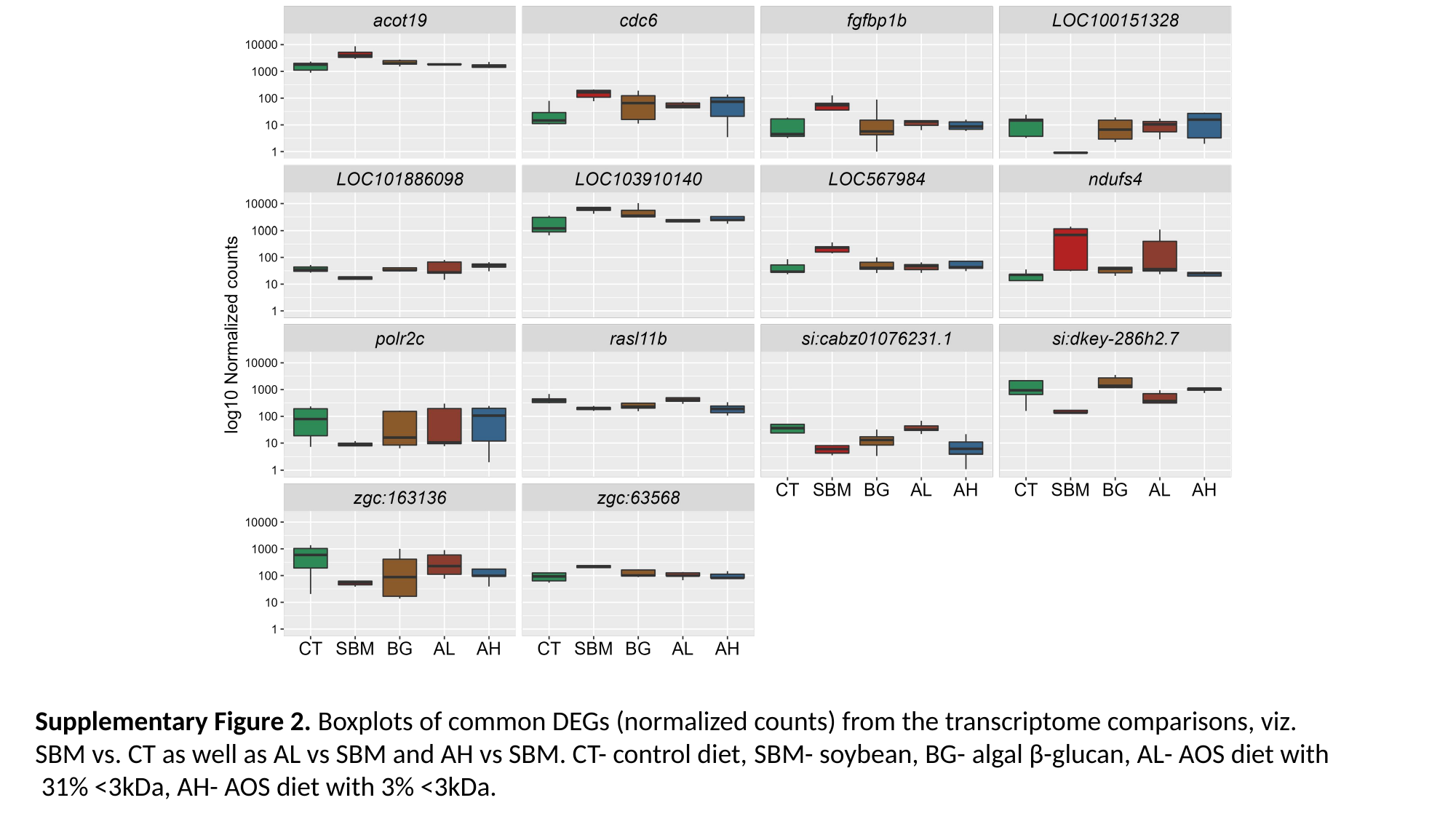

Supplementary Figure 2. Boxplots of common DEGs (normalized counts) from the transcriptome comparisons, viz.
SBM vs. CT as well as AL vs SBM and AH vs SBM. CT- control diet, SBM- soybean, BG- algal β-glucan, AL- AOS diet with
 31% <3kDa, AH- AOS diet with 3% <3kDa.

## Slide 3
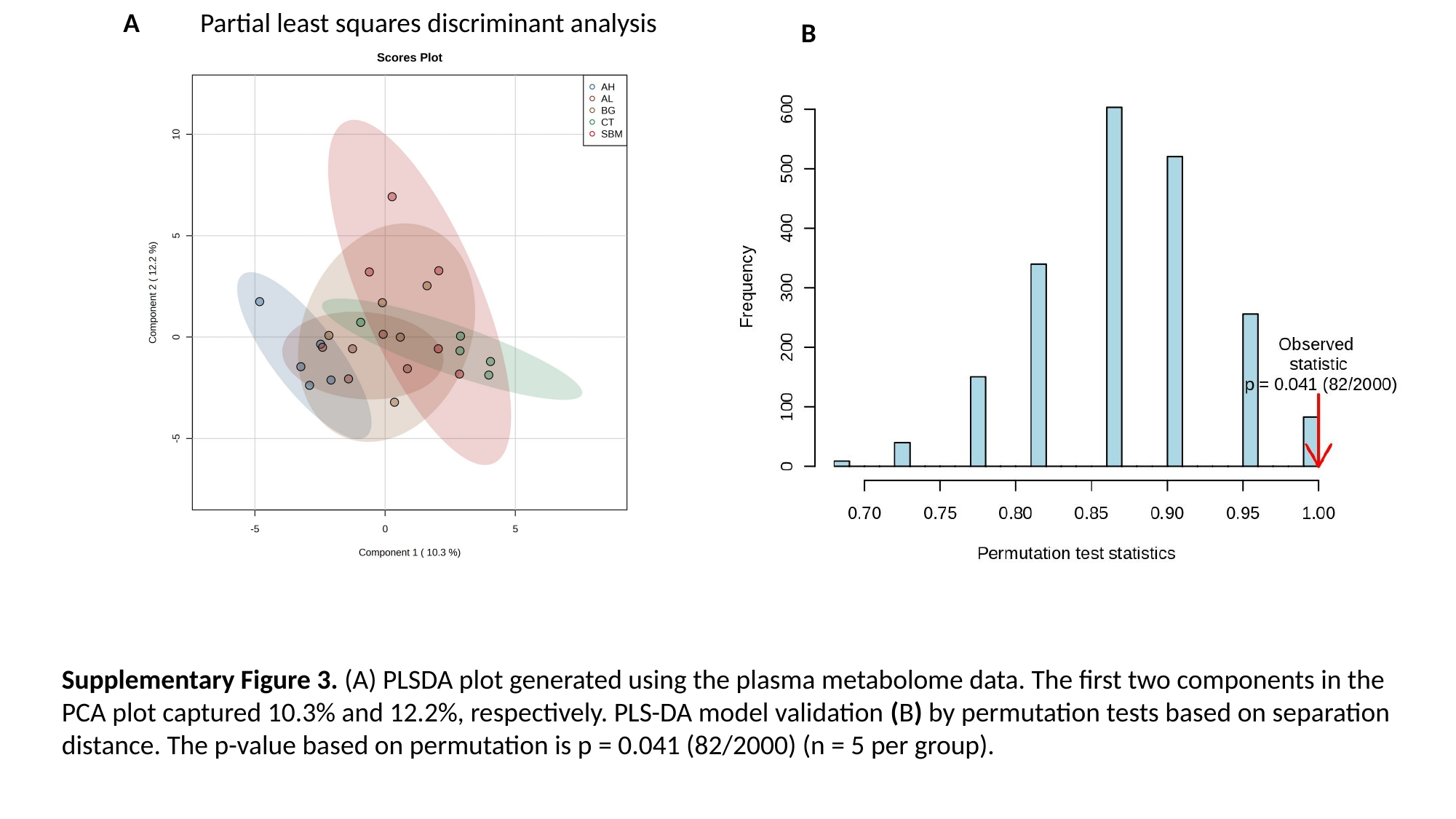

A
Partial least squares discriminant analysis
B
Supplementary Figure 3. (A) PLSDA plot generated using the plasma metabolome data. The first two components in the
PCA plot captured 10.3% and 12.2%, respectively. PLS-DA model validation (B) by permutation tests based on separation
distance. The p-value based on permutation is p = 0.041 (82/2000) (n = 5 per group).

## Slide 4
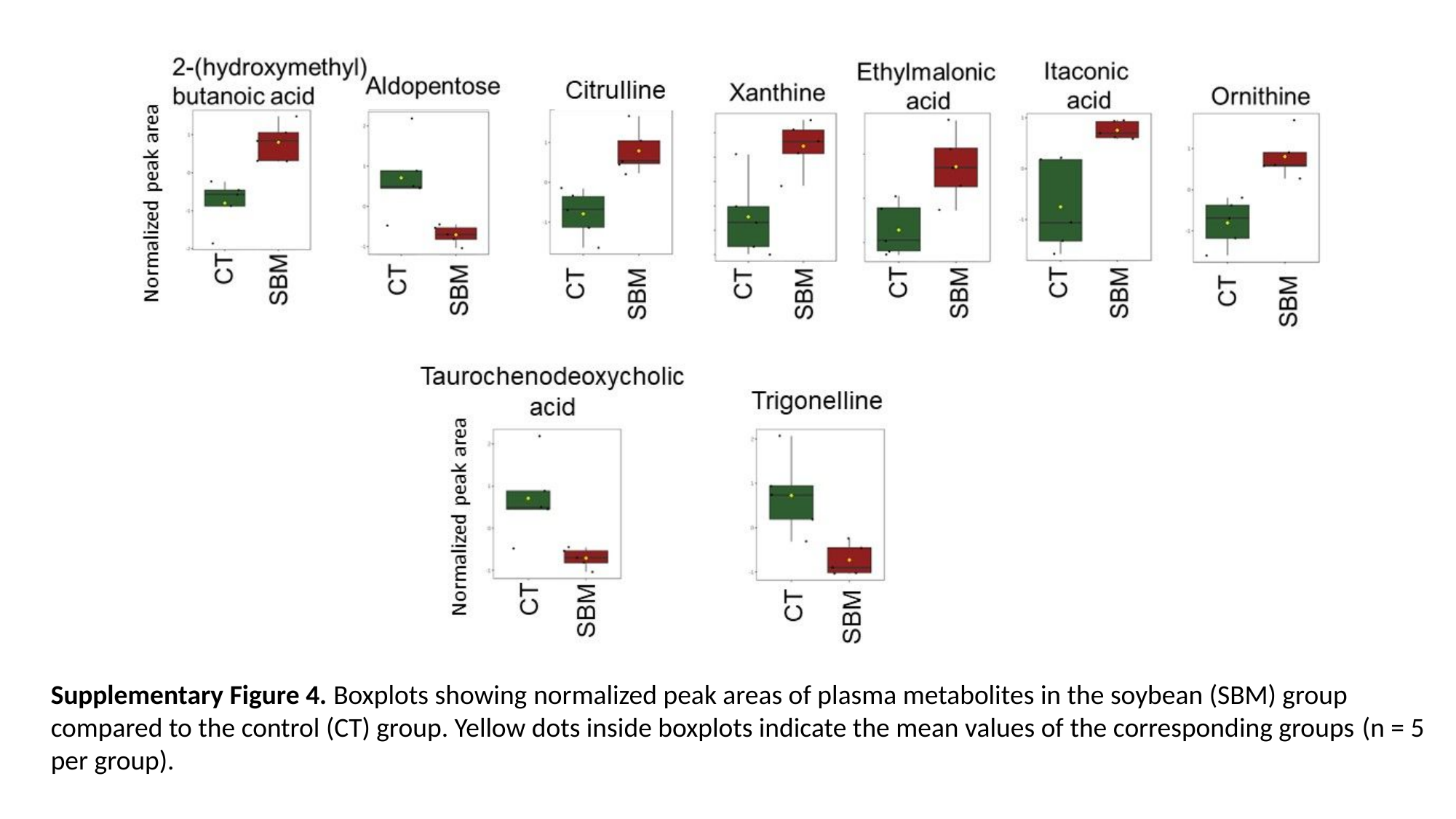

Supplementary Figure 4. Boxplots showing normalized peak areas of plasma metabolites in the soybean (SBM) group compared to the control (CT) group. Yellow dots inside boxplots indicate the mean values of the corresponding groups (n = 5 per group).

## Slide 5
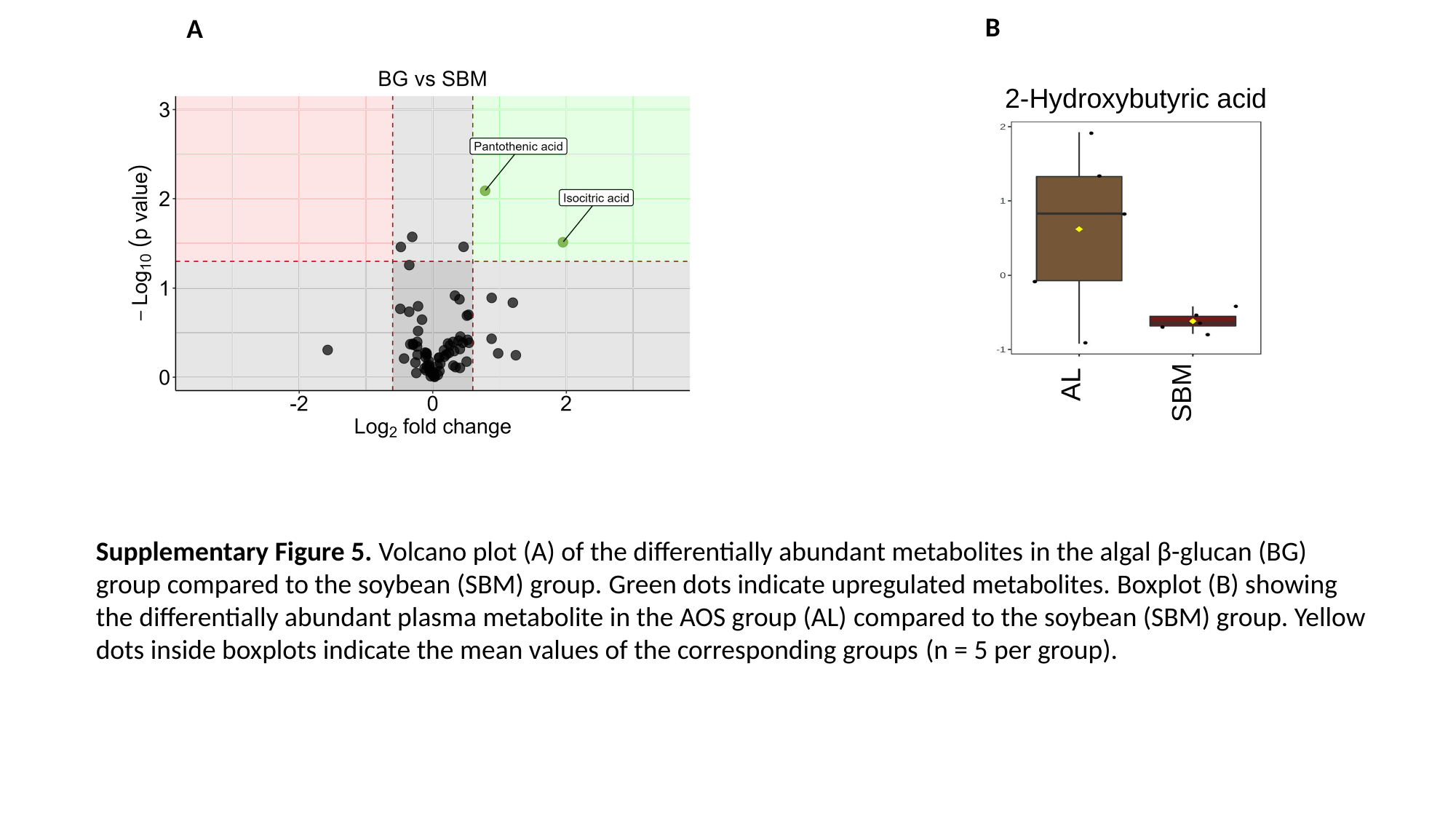

B
A
2-Hydroxybutyric acid
AL
SBM
Supplementary Figure 5. Volcano plot (A) of the differentially abundant metabolites in the algal β-glucan (BG) group compared to the soybean (SBM) group. Green dots indicate upregulated metabolites. Boxplot (B) showing the differentially abundant plasma metabolite in the AOS group (AL) compared to the soybean (SBM) group. Yellow dots inside boxplots indicate the mean values of the corresponding groups (n = 5 per group).
